# Knowledge, attitudes, and practices of primary healthcare practitioners regarding pharmacist clinics: a cross-sectional study in Shanghai

**DOI:** 10.1186/s12913-024-11136-3

**Published:** 2024-05-29

**Authors:** Xinyue Zhang, Zhijia Tang, Yanxia Zhang, Wai Kei Tong, Qian Xia, Bing Han, Nan Guo

**Affiliations:** Minhang Hospital & Department of Clinical Pharmacy, School of Pharmacy, Fudan University, 170 Xinsong Road, Shanghai, 201199 P.R. China

**Keywords:** Pharmacist clinics, Primary care, Cross-sectional study, Pharmaceutical services, Public health

## Abstract

**Background:**

Pharmacist clinics offer professional pharmaceutical services that can improve public health outcomes. However, primary healthcare staff in China face various barriers and challenges in implementing such clinics. To identify existing problems and provide recommendations for the implementation of pharmacist clinics, this study aims to assess the knowledge, attitudes, and practices of pharmacist clinics among primary healthcare providers.

**Methods:**

A cross-sectional survey based on the Knowledge-Attitude-Practice (KAP) model, was conducted in community health centers (CHCs) and private hospitals in Shanghai, China in May, 2023. Descriptive analytics and the Pareto principle were used to multiple-answer questions. Chi-square test, Fisher’s exact test, and binary logistic regression models were employed to identify factors associated with the knowledge, attitudes, and practices of pharmacist clinics.

**Results:**

A total of 223 primary practitioners participated in the survey. Our study revealed that most of them had limited knowledge (60.1%, *n* = 134) but a positive attitude (82.9%, *n* = 185) towards pharmacist clinics, with only 17.0% (*n* = 38) having implemented them. The primary goal of pharmacist clinics was to provide comprehensive medication guidance (31.5%, *n* = 200), with medication education (26.3%, *n* = 202) being the primary service, and special populations (24.5%, *n* = 153) identified as key recipients. Logistic regression analysis revealed that education, age, occupation, position, work seniority, and institution significantly influenced their perceptions. Practitioners with bachelor’s degrees, for instance, were more likely than those with less education to recognize the importance of pharmacist clinics in medication guidance (aOR: 7.130, 95%CI: 1.809–28.099, *p*-value = 0.005) and prescription reviews (aOR: 4.675, 95% CI: 1.548–14.112, *p*-value = 0.006). Additionally, practitioners expressed positive attitudes but low confidence, with only 33.3% (*n* = 74) feeling confident in implementation. The confidence levels of male practitioners surpassed those of female practitioners (*p*-value = 0.037), and practitioners from community health centers (CHCs) exhibited higher confidence compared to their counterparts in private hospitals (*p*-value = 0.008). Joint physician-pharmacist clinics (36.8%, *n* = 82) through collaboration with medical institutions (52.0%, *n* = 116) emerged as the favored modality. Daily sessions were preferred (38.5%, *n* = 86), and both registration and pharmacy service fees were considered appropriate for payment (42.2%, *n* = 94). The primary challenge identified was high outpatient workload (30.9%, *n* = 69).

**Conclusions:**

Although primary healthcare practitioners held positive attitudes towards pharmacist clinics, limited knowledge, low confidence, and high workload contributed to the scarcity of their implementation. Practitioners with diverse sociodemographic characteristics, such as education, age, and institution, showed varying perceptions and practices regarding pharmacist clinics.

**Supplementary Information:**

The online version contains supplementary material available at 10.1186/s12913-024-11136-3.

## Background

Pharmacist clinics are specialized healthcare facilities that offer professional pharmaceutical services, such as medication therapy management, medication reconciliation, lifestyle counseling, and immunizations, for patients with chronic diseases or managing multiple drugs [1]. Through the provision of these services, pharmacist clinics aim to improve patient access to healthcare, optimize medication use, and improve overall public health outcomes.

Pharmacist clinics originated in the 1960s in the United States and have spread globally in recent decades [2], with a growing number of countries adopting this model of care. The World Health Organization (WHO) has recognized the importance of pharmacists in primary healthcare and encouraged the integration of pharmaceutical services into broader healthcare systems [3]. This integration facilitates the rational use of medication, thereby minimizing adverse drug events and medication errors, ultimately leading to better therapeutic outcomes. Moreover, pharmacist clinics offer medication guidance and education, which adjusts optimal medication dosage [4], enhances patient adherence [1, 5], expands access to health care [6], and reduces treatment costs [7]. These clinics effectively bridge the communication gap between physicians and pharmacists [8], fostering interdisciplinary collaboration and integrated patient care [1, 9].

The development of pharmacist clinics in China was initiated in the late 20th century, coinciding with the introduction of healthcare reforms by the Chinese government in the early 2000s. The release of “Opinions on Deepening the Reform of the Medical and Health System” [10] in 2009 highlighted the importance of pharmacist clinics and the crucial role of pharmacists in improving the quality and accessibility of healthcare services in primary settings. In 2020, the Chinese government released a guidance document titled “Opinions on Strengthening the Pharmaceutical Management of Medical Institutions and Promoting Rational Drug Use,” encouraging provinces to actively establish pharmacist clinics [11]. However, it wasn’t until 2021 that the General Office of the National Health Commission developed the “Guidelines for Pharmaceutical Outpatient Services in Medical Institutions” to standardize these pharmacist clinics [12]. Despite the progress made, primary medical staff in both developed and developing countries face various challenges, especially in developing countries [13], including a shortage of qualified pharmacists [14, 15], limited recognition of pharmacists’ roles among healthcare professionals and the public [16, 17], and the need for a more standardized approach to pharmaceutical care [18]. Additionally, these clinics are predominantly located in large general hospitals or specialized medical facilities, limiting their coverage to specific areas, such as antibiotics [19] and anticoagulants [20]. In rural areas, there is scarce awareness and discussion regarding the promotion of pharmacist clinics.

To date, most research on pharmacist clinics comes from countries like the United States, the UK, Canada, and Australia, focusing primarily on the outcomes of pharmacist interventions rather than the implementation challenges [1, 4, 21–24]. In China, only a few studies have assessed the current state of pharmacist clinics. Cai et al. [25], for instance, conducted a national survey revealing that just 10.03% of hospitals had pharmacist clinics. Wu et al. [26] investigated the establishment and operational details of pharmacist-managed clinics in Taiwan. However, there is no published research exploring optimal practices for setting up pharmacist clinics in China or identifying the barriers to establishing these clinics in primary healthcare settings. In this study, we aim to assess the awareness and understanding of pharmacist clinics among primary healthcare providers. We conducted a cross-sectional survey based on the Knowledge-Attitude-Practice (KAP) model to identify knowledge gaps and develop interventions to encourage interprofessional collaboration and enhance practice efficiency. The findings may also improve patient outcomes, healthcare delivery by streamlining the implementation process, and utilization of high-quality pharmaceutical services. Our ultimate goal was to overcome barriers to advancing pharmacist clinics within China’s healthcare system and offer insights for policymakers and healthcare authorities to integrate these clinics into primary healthcare settings, not only in China but potentially in other countries as well.

## Methods

### Survey instrument & selection criteria

Our study employed a structural equation model based on the Knowledge, Attitude, and Practice (KAP) theory [27] and relevant literature [28–31] to explore the relationships between various factors. Following the KAP principles, we developed a questionnaire consisting of 21 questions across three domains: (A) knowledge of pharmacist clinics, (B) attitudes towards pharmacist clinics, and (C) practices related to pharmacist clinics. Demographic information such as gender, age, education, occupation, position, seniority, department, and institution was collected through self-reporting.

The inclusion and exclusion criteria for the sampled respondents were as follows. Inclusion criteria: (1) Full-time primary healthcare practitioners attending a continuing education course at Minhang Hospital in Shanghai, China. This included physicians, pharmacists, nurses, and other primary healthcare practitioners. (2) Willingness to participate in the study and provide informed consent. Exclusion criteria: (1) Part-time employees or interns. (2) Non-medical staff. (3) Individuals who declined to sign the informed consent form.

### Study population and data source

This study used data from a cross-sectional survey conducted in May, 2023, involving primary healthcare practitioners from 10 community health centers (CHCs) and 38 private hospitals in Shanghai, China. After excluding participants from secondary or tertiary hospitals (*n* = 9), nursing homes (*n* = 6), and other facilities such as welfare homes and school clinics (*n* = 9), a total of 223 eligible subjects were included.

### Data collection

The sample size was optimized to range between 105 and 210, based on the recommended ratio of 5 to 10 respondents per item [32, 33]. We also performed a pilot study in April, 2023 to ensure linguistic clarity and readability of the questionnaire. Twenty-six student volunteers from the School of Pharmacy at Fudan University were recruited to refine the questionnaire. Additionally, face-to-face interviews were conducted to further assess their understanding of the content. The final version was electronically distributed to participants during a continuing education course using a voluntary sampling approach. The full questionnaire is available in Supplementary Table 1, and all data were anonymized.

### Statistical analysis

Categorical variables were summarized using frequency counts (weighted percentage, %). The Chi-square test and Fisher’s exact test were used to assess differences in knowledge, attitude, and practice regarding pharmacist clinics across various sociodemographic characteristics. Descriptive analytics and the Pareto principle were applied to multiple-answer questions. In case of rejection of the null hypothesis, multiple pairwise comparisons would be conducted as confirmatory post hoc analysis using Bonferroni correction. Based on the univariate analysis results, we constructed binary logistic regression models to calculate adjusted odds ratios (aOR) and 95% confidence intervals (CI) to reveal factors associated with perceived goals, service scope, and target recipients of pharmacist clinics.

All statistical analyses were performed using IBM SPSS Statistics for Windows, Version 20.0 (IBM Corp., Armonk, NY, USA). A two-sided *p*-value < 0.05 was considered statistically significant.

## Results

### Demographics

As presented in Table 1, a total of 223 primary healthcare practitioners participated in the survey, with 41.3% (*n* = 92) being male and 76.2% (*n* = 170) under 45 years old. The majority (84.3%, *n* = 188) were physicians, while the remaining were pharmacists. Regarding educational qualifications, 82.5% (*n* = 184) of respondents held a bachelor’s degree or below. Furthermore, 91.9% (*n* = 205) held mid-level or lower positions, and 56.1% (*n* = 125) reported professional tenures of less than 10 years. Of these 223 practitioners, 36.8% (*n* = 82) were from public institutions (community health centers), and 63.2% (*n* = 141) were from private hospitals.

**Table 1:** Sociodemographic information of respondents (*N* = 223)

Variables	Characteristics	Frequency (*n*)	Percentage (%)
Gender	Male	92	41.3
	Female	131	58.7
Age, years	18–30	68	30.5
	31–45	102	45.7
	> 45	53	23.8
Education	Junior college or below	48	21.5
	Bachelor’s degree	136	61.0
	Master degree or above	39	17.5
Occupation	Physician	188	84.3
	Pharmacist	35	15.7
	Nurse	0	0.0
	Other	0	0.0
Department	Internal medicine	52	23.3
	Surgery	36	16.1
	General practice	39	17.5
	Traditional Chinese medicine	27	12.1
	Pharmacy	35	15.7
	Other	34	15.2
Work seniority, years	< 5	59	26.5
	5–9	66	29.6
	10–19	39	17.5
	≥ 20	59	26.5
Position	Senior/deputy senior	18	8.1
	Intermediate	97	43.5
	Junior or below	108	48.4
Institution	Community health center	82	36.8
	Private hospital	141	63.2

### Knowledge of pharmacist clinics

**Fig. 1:**
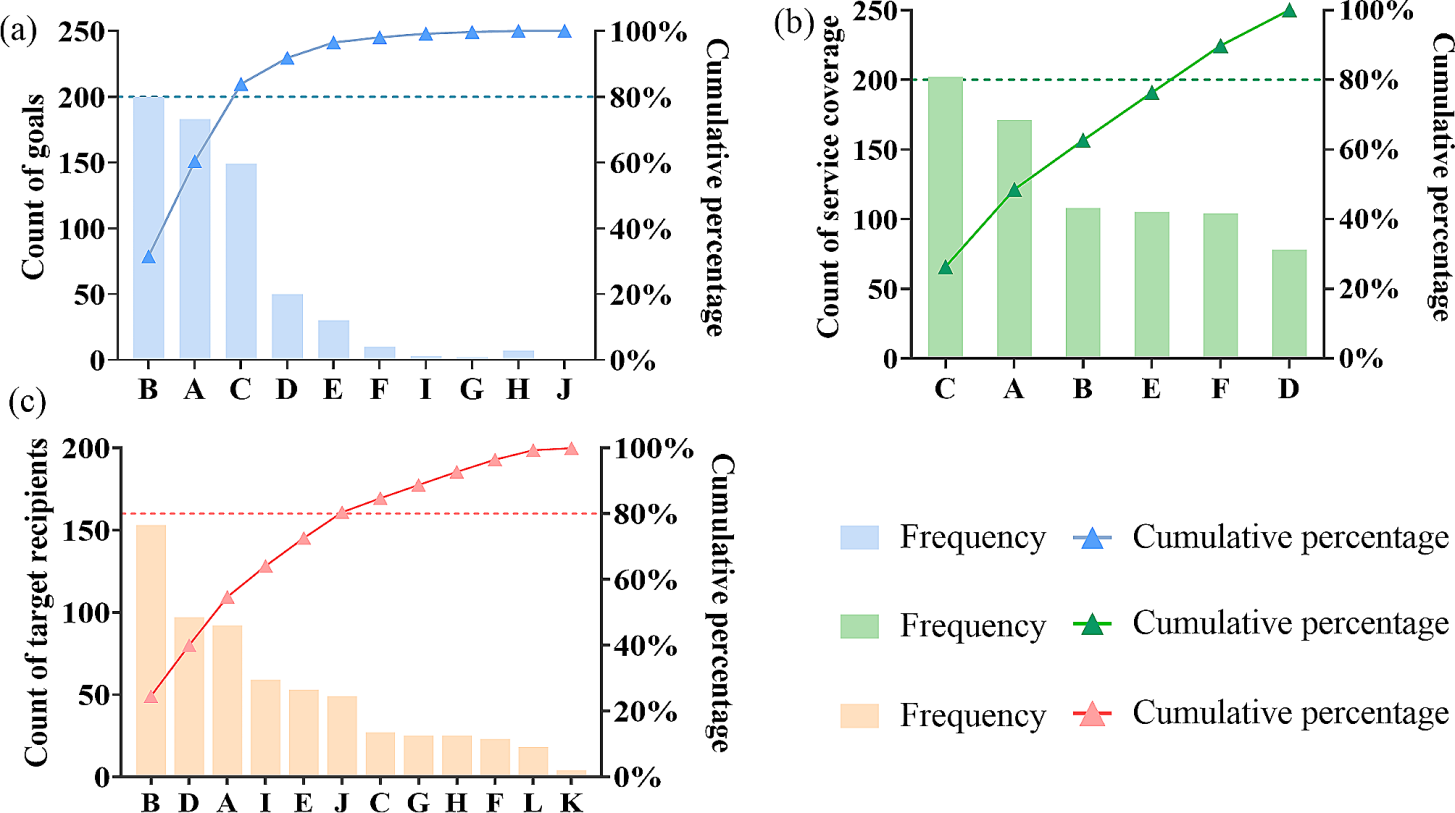
Pareto chart demonstrating respondents’ knowledge of pharmacist clinics (**a**) Perceived goals: **A** prescription reviews, **B** medication guidance, **C** time-saving, **D** conflict alleviation, **E** patient empowerment, **F** cost reduction, **G** role enhancement, **H** research, **I** training, and **J** no perceived value (**b**) Perceived service scope: **A** drug regimen adjustments, **B** medication reconciliation, **C** medication education on dosage, side effects, and interactions, **D** adherence interventions, **E** health promotion, **F** patient follow-ups (**c**) Perceived target recipients: **A** isolated/empty-nest patients, **B** special populations (e.g. elderly, children, pregnant, and liver/kidney-impaired), **C** economically disadvantaged patients, **D** patients suffering from adverse reactions, **E** patients needing test report interpretations, **F** frequent drug collectors (> 20 times/year), **G** patients with ≥ 2 chronic diseases, **H** patients with any chronic diseases, **I** patients on ≥ 5 medications, **J** high-risk drug users (e.g. psychotropic drugs, hormones, injections, and inhalants), **K** patients under contract with family physicians, and **L** all patients

Of primary care practitioners, 84.8% (*n* = 189) recognized pharmacist clinics, with 24.7% (*n* = 55) having strong familiarity. Figure 1a-c showed practitioners’ views on the goals, services, and target recipients of these clinics. The primary goal was to provide comprehensive medication guidance (31.5%, *n* = 200), with medication education (26.3%, *n* = 202) being the primary service, and special populations (24.5%, *n* = 153) identified as key recipients. Logistic regression results revealed several significant influential factors (Table 2).

**Table 2:** Factors influencing perceived goals, service scope, and target recipients of pharmacist clinics (*N* = 223)

Factors	Reference	Characteristics	Items		Odds Ratio (95% CI)	Adjusted *p*-value	Hosmer-Lemeshow Good of Fit test	
							Chi-square	*p*-value
Education	Junior college or below	Bachelor	Goal	Prescription reviews	4.675 (1.548–14.112)	0.006	6.273	0.617
				Medication guidance	7.130 (1.809–28.099)	0.005	7.144	0.521
			Recipient	Patients needing test report interpretations	0.384 (0.159–0.928)	0.034	3.899	0.866
				High-risk drug users	2.824 (1.090–7.316)	0.033	6.730	0.566
		Master or above	Goal	Medication guidance	7.303 (1.343–39.720)	0.021	7.144	0.521
			Service	Adherence interventions	4.221 (1.339–13.300)	0.014	11.443	0.178
				Follow-up visits	3.125 (1.095–8.915)	0.033	5.486	0.705
			Recipient	Patients needing test report interpretations	0.126 (0.032–0.505)	0.003	3.899	0.866
				Patients with ≥ 2 chronic diseases	6.401 (1.233–33.223)	0.027	7.581	0.475
				Patients on ≥ 5 medications	3.987 (1.250-12.717)	0.019	5.98	0.649
Age	18–30 years old	≥ 46 years old	Goal	Conflict alleviation	0.165(0.028–0.988)	0.048	5.618	0.690
Occupation	Physician	Pharmacist	Service	Drug regimen adjustments	0.210 (0.088-0.500)	< 0.001	3.826	0.873
			Recipient	All patients	3.322 (1.031–10.703)	0.044	6.131	0.633
Position	Senior/deputy senior	Intermediate	Service	Follow-up visits	3.990 (1.087–14.646)	0.037	5.486	0.705
			Recipient	Patients needing test report interpretations	0.287 (0.082–0.997)	0.049	3.899	0.866
		Junior or below	Service	Follow-up visits	5.832 (1.308–25.998)	0.021	5.486	0.705
			Recipient	Patients needing test report interpretations	0.172 (0.038–0.781)	0.023	3.899	0.866
				High-risk drug users	18.391 (1.686-200.604)	0.017	6.730	0.566
Work seniority	< 5 years	10–19 years	Recipient	Isolated/empty-nest patients	3.328 (1.021–10.849)	0.046	5.209	0.735
Institution	Community health center	Private hospital		Frequent drug collectors	0.359 (0.134–0.966)	0.043	5.666	0.685

The model incorporated variables including gender, age, education, occupation, position, work seniority, and institution. The variable “department” was excluded due to inadequate model fit. Only significant findings were presented

#### Education

Compared to those with less education, practitioners with bachelor’s degrees were more likely to see the role of pharmacist clinics in medication guidance (aOR: 7.130, 95%CI: 1.809–28.099, *p*-value = 0.005), prescription reviews (aOR: 4.675, 95% CI: 1.548–14.112, *p*-value = 0.006), and serving patients on high-risk drugs (aOR: 2.824, 95% CI: 1.090–7.316, *p*-value = 0.033).

Besides medication guidance (aOR: 7.303, 95%CI: 1.343–39.720, *p*-value = 0.021), practitioners with master’s or higher degrees preferred adherence interventions (aOR: 4.221, 95%CI: 1.339–13.300, *p*-value = 0.014), follow-up services (aOR: 3.125, 95%CI: 1.095–8.915, *p*-value = 0.033), and catering to patients with ≥ 2 chronic diseases (aOR: 6.401, 95%CI: 1.233–33.223, *p*-value = 0.027) or ≥ 5 medications (aOR: 3.987, 95%CI: 1.250-12.717, *p*-value = 0.019). Higher education was also inversely associated with emphasizing patients needing test report interpretations (aOR < 1, *p*-value < 0.05).

#### Age

Younger practitioners, aged 18 to 30, considered pharmacist clinics as tools to mitigate physician-patient conflicts through improved communication compared to those aged ≥ 46 (aOR: 0.165, 95%CI: 0.028–0.988, *p*-value = 0.048).

#### Occupation

Compared to physicians, pharmacists typically addressed all patients as recipients (aOR: 3.322, 95%CI: 1.031–10.703, *p*-value = 0.044), but were less likely to offer drug regimen adjustments (aOR: 0.210, 95%CI: 0.088-0.500, *p*-value < 0.001).

#### Position

Junior and intermediate-level practitioners demonstrated a greater likelihood for follow-up services (aOR_1_: 5.832, 95%CI: 1.308–25.998, *p*-value = 0.021; aOR_2_: 3.99, 95%CI: 1.087–14.646, *p*-value = 0.037), and were less likely to target patients in need of test report interpretations (aOR_1_: 0.172, 95%CI: 0.038–0.781, *p*-value = 0.023; aOR_2_: 0.287, 95%CI: 0.082–0.997, *p*-value = 0.049) than their senior counterparts.

#### Work seniority

Practitioners with 10–19 years of work experience were significantly more likely to consider isolated/empty-nest patients as suitable recipients compared to those with < 5 years of experience (aOR: 3.328, 95%CI: 1.021–10.849, *p*-value = 0.046).

#### Institution

Practitioners from CHCs were more likely to view frequent drug collectors as suitable recipients compared to those from private hospitals (aOR: 0.359, 95%CI: 0.134–0.966, *p*-value = 0.043).

### Attitude of pharmacist clinics

#### Necessity and confidence in implementing pharmacist clinics

Table 3 showed that 82.9% (*n* = 185) of practitioners recognized the necessity of pharmacist clinics, but only 33.3% (*n* = 75) felt confident in their implementation. Male practitioners exhibited significantly higher confidence levels compared to female practitioners (*p* = 0.037), and practitioners from community health centers (CHCs) showed greater confidence relative to those practicing in private hospitals (*p* = 0.008).

**Table 3:** Primary healthcare practitioners’ attitude toward conducting pharmacist clinics, n (%)

Items	Totally agree	Agree	Uncertain	Disagree	Totally disagree	*P*-value
Necessity	96 (43.0)	89 (39.9)	36 (16.1)	2 (0.9)	0 (0.0)	*
Confidence	23 (10.3)	52 (23.3)	81 (36.3)	53 (23.8)	14 (6.3)	
*Gender*						0.037
Male	11 (12.0)	24 (26.1)	35 (38.0)	13 (14.1)	9 (9.8)	
Female	12 (9.2)	28 (21.4)	46 (35.1)	40 (30.5)	5 (3.8)	
*Institution*						0.008
CHC	13 (15.9)	26 (31.7)	27 (32.9)	14 (17.1)	2 (2.4)	
Private hospital	10 (7.1)	26 (18.4)	54 (38.3)	39 (27.7)	12 (8.5)	

*No significant differences were observed among all subgroups

#### Preferred mode of pharmacist clinics

As shown in Table 4, the favored modality was found to be joint physician-pharmacist clinics (36.8%, *n* = 82), through collaboration with medical institutions (52.0%, *n* = 116). Daily sessions emerged as the preferred frequency (*n* = 86, 38.5%), with both registration and pharmacy service fees considered appropriate for payment (42.2%, *n* = 94).

Furthermore, we explored the influence of different sociodemographic variables. Practitioners holding a master’s degree or higher demonstrated a preference for a clinic frequency of 2–4 times per week (*p*-value = 0.015), along with acceptance of both registration and pharmacy service fees (*p*-value < 0.001), compared to those with lower levels of education. Conversely, those with a junior college education or below were more willing to seek free services. Practitioners from CHCs exhibited a preference for weekly or 2–4 times per week clinics, whereas those from private hospitals favored daily or monthly sessions (*p*-value < 0.001).

**Table 4:** Correlation of preferred mode of pharmacist clinics with education and institution

Items	Contents	Education			*P*-value	Institution		*P*-value
		Junior college or below	Bachelor	Master or above		Community health center	Private hospital	
Modality	Physician-guided traditional clinic	14 (29.2)	31 (22.8)	9 (23.1)	0.751	22 (26.8)	32 (22.7)	0.848
	Independent pharmacist clinic	2 (4.2)	12 (8.8)	1 (2.6)		5 (6.1)	10 (7.1)	
	Joint physician-pharmacist clinic	15 (31.2)	49 (36.0)	18 (46.2)		31 (37.8)	51 (36.2)	
	Multidisciplinary clinic involving physicians, pharmacists, nurses, and nutritionists	15 (31.2)	40 (29.4)	11 (28.2)		23 (28.0)	43 (30.5)	
	Lectures/consultation sessions; no scheduled clinics	2 (1.2)	4 (2.9)	0 (0.0)		1 (1.2)	5 (3.5)	
Approach	Collaboration with research institutions	9 (18.8)	24 (17.6)	8 (20.5)	0.315	19 (23.2)	22 (15.6)	0.309
	Collaboration with medical institutions	20 (41.7)	70 (51.5)	26 (66.7)		41 (50.0)	75 (53.2)	
	Collaboration with enterprises	4 (8.3)	10 (7.4)	2 (5.1)		3 (3.7)	13 (9.2)	
	Collaboration with industry associations	12 (25.0)	19 (14.0)	2 (5.1)		11 (13.4)	22 (15.6)	
	Independent operation	3 (6.2)	12 (8.8)	1 (2.6)		8 (9.8)	8 (5.7)	
	Other	0 (0.0)	1 (0.7)	0 (0.0)		0 (0.0)	1 (0.7)	
Frequency	Daily	16_a_ (33.4)	60_a_ (44.2)	10_a_ (25.7)	0.015	21 (25.6)	65 (46.1)	< 0.001
	2–4 times per week	10_a, b_ (20.8)	24_b_ (17.6)	17_a_ (43.6)		25_a_ (30.5)	26_b_ (18.4)	
	Weekly	12_a_ (25.0)	35_a_ (25.7)	11_a_ (28.2)		32_a_ (39.0)	26_b_ (18.4)	
	Once every two weeks	4_a_ (8.3)	3_a_ (2.2)	1_a_ (2.6)		2_a_ (2.4)	6_a_ (4.3)	
	Monthly	5_a_ (10.4)	13_a_ (9.6)	0_a_ (0.0)		2_a_ (2.4)	16_b_ (11.3)	
	Other (please specify ____)	1_a_ (2.1)	1_a_ (0.7)	0_a_ (0.0)		0_a_ (0.0)	2_a_ (1.4)	
Payment	Registration and pharmacy service fees	12_a_ (25.0)	57_a_ (41.9)	25_b_ (64.1)	< 0.001	36 (43.4)	58 (41.1)	0.634
	Only registration fees determined by provider positions	6_a_ (12.5)	18_a_ (13.2)	6_a_ (15.4)		11 (13.3)	19 (13.5)	
	Only registration fees determined by institution levels	15_a_ (31.2)	47_a_ (34.6)	7_a_ (17.9)		27 (33.7)	42 (29.8)	
	No fee should be charged	15_a_ (31.2)	14_b_ (10.3)	1_b_ (2.6)		8 (9.6)	22 (15.6)	

Each subscript letter denotes a subset of education-level categories whose column proportions do not differ significantly from each other at the 0.05 level

### Practice of pharmacist clinics

As shown in Table 5, there was a limited prevalence of pharmacist clinics within primary care institutions. Only 17.0% (*n* = 38) of practitioners reported the implementation of pharmacy clinics, mostly scheduled once a week (47.4%, *n* = 18), with the primary challenge being a high outpatient workload (30.9%, *n* = 69). Practitioners from CHCs demonstrated a significantly higher implementation frequency compared to those from private hospitals (*p*-value < 0.001).

We further explored sociodemographic factors associated with challenges. Practitioners aged over 45 years (*P* = 0.020) and occupying senior/deputy senior positions (*p*-value = 0.018) were more likely to consider the absence of fee collection mechanisms as the principal difficulty, as opposed to their younger counterparts and those in lower positions.

**Table 5:** Correlation of practice of pharmacist clinics with age and position

Items	Contents	Age, years			*P*-value	Position			*P*-value
		18–30	31–45	> 45		Senior/deputy senior	Intermediate	Junior or below	
Frequency	Daily	3 (4.4)	4 (3.9)	0 (0.0)	0.782	0 (0.0)	5 (5.2)	2 (1.9)	0.716
	2–4 times per week	1 (1.5)	3 (2.9)	1 (1.9)		1 (5.6)	3 (3.1)	1 (0.9)	
	Weekly	4 (5.9)	8 (7.8)	6 (11.3)		2 (11.1)	7 (7.2)	9 (8.3)	
	Once every two weeks	1 (1.5)	1 (1.0)	1 (1.9)		0 (0.0)	2 (2.1)	1 (0.9)	
	Monthly	0 (0.0)	3 (2.9)	1 (1.9)		0 (0.0)	1 (1.0)	3 (2.8)	
	Other	1 (1.5)	0(0.0)	0 (0.0)		0 (0.0)	1 (1.0)	0 (0.0)	
Challenges	Insufficient professionalism	15_a_ (22.1)	30_a_ (29.4)	11_a_ (20.8)	0.020	5_a_ (27.8)	23_a_ (23.7)	28_a_ (25.9)	0.018
	High outpatient workload	24_a_ (35.3)	29_a_ (28.4)	16_a_ (30.2)		2_a_ (11.1)	28_a_ (28.9)	39_a_ (36.1)	
	Limited patient volume	14_a_ (20.6)	24_a_ (23.5)	11_a_ (20.8)		5_a_ (27.8)	27_a_ (27.8)	17_a_ (15.7)	
	Lack of leadership attention	0_a_ (0.0)	3_a_ (2.9)	2_a_ (3.8)		0_a_ (0.0)	2_a_ (2.1)	3_a_ (2.8)	
	Weak inter-department collaboration	1_a_ (1.5)	5_a_ (4.9)	1_a_ (1.9)		1_a_ (5.6)	4_a_ (4.1)	2_a_ (1.9)	
	Space constraints	5_a_ (7.4)	6_a_ (5.9)	1_a_ (1.9)		1_a_ (5.6)	3_a_ (3.1)	8_a_ (7.4)	
	Absence of fee collection mechanisms	1_a_ (1.5)	1_a_ (1.0)	8_b_ (15.1)		2_a_ (11.1)	7_a, b_ (7.2)	1_b_ (0.9)	
	Low staff motivation	8_a_ (11.8)	4_a_ (3.9)	2_a_ (3.8)		1_a_ (5.6)	3_a_ (3.1)	10_a_ (9.3)	
	No significant difficulties identified	0_a_ (0.0)	0_a_ (0.0)	1_a_ (1.9)		1_a_ (5.6)	0_a, b_ (0.0)	0_b_ (0.0)	

Each subscript letter denotes a subset of education-level categories that do not differ significantly from each other at the 0.05 level. Only statistically significant results were presented

## Discussion

Our study aims to evaluate the perceptions, attitudes, and practices of primary healthcare practitioners regarding pharmacist clinics and to identify necessary changes. The findings unveiled a lack of knowledge and confidence among primary care providers, who are faced with barriers including high outpatient workloads and concerns related to professionalism. Collaborative models are preferred as they align with the current emphasis on multidisciplinary approaches in modern healthcare, which aim to achieve optimal population health [34]. Additionally, our findings highlight the impact of institution and gender on the perceptions of primary care providers.

In this study, more practitioners preferred joint physician-pharmacist clinics over traditional physician-led clinics (36.8%, *n* = 82 vs. 24.2%, *n* = 54), which is in line with a global focus on integrating pharmacists into the provision of patient-centered, coordinated, and comprehensive care [1, 35, 36]. Primary care physicians are in short supply, and studies unveiled that the shortage of primary care physicians has led to increased workloads and a greater demand for medication guidance services, especially among vulnerable patients aged 65 and above [37–40]. Our study showed the primary goals of pharmacist clinics were found to be prescription reviews (28.9%, *n* = 183) and medication guidance (31.5%, *n* = 200), which are critical in addressing concerns regarding poorly managed or duplicate prescriptions [41, 42]. Integrating pharmaceutical services into primary care offers expedited access and convenience for patients, thereby releasing physicians to focus on more complex cases and reducing their workload [43, 44]. These services also contribute to overall savings in healthcare and medication costs, as well as reduced general physician appointments, emergency department visits, and inappropriate drug use [45, 46]. Our findings support the potential of pharmacist-led prescription reviews in reducing duplicate prescriptions [47], drug-related problems [48], and medication costs, without increasing physicians’ workload [49]. Moreover, pharmacist-led medication guidance provided to other professionals has been shown to reduce medication errors and inappropriate prescriptions compared to standard care [50, 51]. The development of joint physician-pharmacist clinics may be an advantageous choice for the development of pharmacist clinics in the future.

Current evidence highlights the suboptimal quality of primary care in China [52], with previous research suggesting that inadequate education and training pose significant challenges in enhancing care quality [53]. Primary healthcare providers in China have reported being too busy for continued education, dissatisfaction with course content, and having unqualified supervisors [54]. This issue seems to be consistent in the United States [55], Canada [56], and Belgium [57]. Moreover, our study has identified high workload (30.9%, *n* = 69) and insufficient professionalism (25.1%, *n* = 56) as the top two challenges faced by pharmacist clinics. On the other hand, insufficient knowledge may contribute to negative attitudes [39].

In this study, a minority of practitioners (24.7%, *n* = 55) demonstrated strong familiarity, and only 33.3% (*n* = 75) felt confident. While some global studies did not find a significant difference in clinical competence confidence between public and private practitioners [58, 59], our study revealed that pharmacists from CHCs exhibited greater confidence in conducting pharmacist clinics compared to those from private hospitals, partially due to their greater exposure to training. Studies have also shown that community pharmacists, through enhanced training, can acquire expanded expertise and knowledge [60, 61], leading to improved service quality in primary care [62, 63]. Future efforts should focus on establishing a more efficient learning and continued education system for community practitioners in China [52].

Several impediments were identified by respondents, including limited patient volume (22.0%, *n* = 49) and low staff motivation (6.3%, *n* = 14). Despite the positive impact of pharmacists in outpatient settings on patient outcomes, the adoption of these services remains low [1]. Recent literature has highlighted public uncertainty about primary care specialties and skepticism regarding their capacity to deliver comprehensive care [64]. Evidence suggests a lack of awareness, demand, and utilization of community pharmacy services among patients [65, 66]. Another barrier is the prevailing focus on quantity rather than quality of care, with job content and bonuses linked more to quantity than the quality of care delivered [52, 67]. Financial conflicts over funding and the absence of fee collection may also hinder collaboration between pharmacists and other healthcare providers [43, 68]. Additionally, the implementation of the zero-mark-up drug policy in China in 2011 caused a substantial decrease of about 40% in drug-related incomes [69]. Institutions responded by scaling back clinical care services to offset this profit loss [70], leading to an uptick in hospital visits for minor ailments and further burdening the healthcare system [53]. It is important to expand community pharmacy services by establishing reimbursement mechanisms to relieve the burden on general practice [71]. Countries like Australia, the UK, New Zealand, and Canada have established systems for pharmacist remuneration [72]. Payment models for pharmaceutical services typically include fee-for-service, where providers are compensated based on the services delivered (as seen in Australia, Canada, Belgium, and Japan), capitation, where providers receive a fixed amount per patient (as in the US, Thailand, and Denmark), and blended funding, which combines government and private payments (as in China, Australia, New Zealand, and Canada) [73]. Despite the existence of various payment models for pharmaceutical services, there is no standardized pricing for pharmacist clinics. Among 465 hospitals with pharmacist clinics, only 98 (21.08%) owned charging mechanisms [25]. Various studies have explored the willingness to pay (WTP) for pharmaceutical services in different countries. For instance, Porteous et al. [74] found a WTP of $69.19 for community practices in the UK. Tsao et al. [75] reported a WTP of $21.26 for medication therapy management in Canada, and in Brazil, the estimated WTP for comprehensive medication management was $17.75 [76].

Our findings also revealed gender-based disparities in the perceptions and implementation of pharmacist clinics. Female practitioners exhibited lower levels of confidence in conducting the clinics compared to males, consistent with previous research indicating that women in healthcare often perceive deficiencies in their abilities despite no differences in clinical performance between genders [77]. Additionally, female medical students reported higher levels of anxiety, stress, and self-doubt about their knowledge and performance [78]. However, in Australia and Ireland, females rated themselves higher than males in self-assessment tests [79, 80]. Further investigations to explore potential confounding factors, such as cultural influences, may contribute to understanding these variations and better address the need to tailor pharmacist-managed clinic services based on institutional needs [81].

This research is geographically confined to Shanghai and solely captures the perspectives of practitioners, potentially limiting generalizability. Future studies should broaden their scope to encompass diverse practices and include patients’ perceptions. The cross-sectional design used in this study restricts the evaluation of cause-effect relationships, emphasizing the need for longitudinal investigations. Despite these limitations, to the best of the authors’ knowledge, this is the first quantitative study that has examined the knowledge, attitudes, and practice of practitioners regarding pharmacist clinics in primary settings based on real-world data in China. The identified challenges in conducting these clinics provide valuable insights for policymakers, researchers, and institutions in this field.

## Conclusion

Although primary healthcare practitioners generally hold positive attitudes towards pharmacist clinics, limited knowledge and confidence, high workload, and other factors lead to the scarcity of such clinics. Practitioners with diverse sociodemographic backgrounds, especially those from different institutions and genders, exhibit varying perceptions of the forms of pharmacist clinics. Further exploration with lager samples from different regions and service recipients is necessary.

### Electronic supplementary material

Below is the link to the electronic supplementary material.

Supplementary Material 1

## Data Availability

The datasets used and/or analyzed during the current study are available from the corresponding author on reasonable request.
