# Supplementary material for: Knowledge, attitudes, and practices of primary healthcare practitioners regarding pharmacist clinics: a cross-sectional study in Shanghai
Source: BMC Health Serv Res. 2024 May 29;24:677. doi: 10.1186/s12913-024-11136-3 (PMC11134695; doi:10.1186/s12913-024-11136-3)
Supplement: Supplementary file 1 — Supplementary Material 1 [file 12913_2024_11136_MOESM1_ESM.pdf]

Title

Knowledge, attitudes, and practices of primary healthcare practitioners regarding pharmacist clinics: a cross-sectional study in Shanghai

Authors' full names and institutions

Xinyue ZHANG[1,#], Zhijia TANG[1,#], Yanxia ZHANG[1,#], Wai Kei TONG[1], Qian XIA[1], Bing HAN[1,*], Nan GUO[1,*]

[1]Minhang Hospital & Department of Clinical Pharmacy, School of Pharmacy, Fudan University, Shanghai, China

[#]The author contributed equally to the work

*Correspondence

Supplementary Table 1. Questionnaire on pharmacist clinics for primary healthcare practitioners in Shanghai of China.

Items	Answers
Q1: Your gender is	A. Male
	B. Female
Q2: Your age is	A. 18-30 years old
	B. 31-45 years old
	C. >45 years old
Q3: Your highest academic degree is	A. Junior college or below
	B. Bachelor's degree
	C. Master degree or above
Q4: Your occupation is	A. Physician
	B. Pharmacist
	C. Nurse
	D. Other (please specify _____)
Q5: Your department is	A. Internal medicine
	B. Surgery
	C. General practice
	D. Traditional Chinese medicine
	E. Pharmacy
	F. Other
Q6: Your seniority as a primary healthcare practitioner is	A. <5 years
	B. 5-9 years
	C. 10-19 years
	D. ≥20 years
Q7: Your job title is	A. Senior/deputy senior
	B. Intermediate
	C. Junior or below
Q8: Your institution of work is	A. Community health center
	B. Private hospital
Q9: Are you familiar with "pharmacist clinics"?	A. Yes, I'm familiar with it.
	B. Yes, but have limited knowledge.
	C. No, I don't know anything about it.
Q10: What do you think is the primary goal of pharmacist clinics? (multiple-answer)	A. Prescription reviews
	B. Medication guidance
	C. Time-saving
	D. Conflict alleviation
	E. Patient empowerment
	F. Cost reduction
	G. Role enhancement
	H. Research
	I. Training
	J. No perceived value
Q11: What do you think is the service scope of pharmacist clinics? (multiple-answer)	A. Drug regimen adjustments
	B. Medication reconciliation
	C. Medication education on dosage, side effects, and interactions
	D. Adherence interventions
	E. Health promotion
	F. Patient follow-ups
Q12: What do you think is the target recipients of pharmacist clinics? (multiple-answer)	A. Isolated/empty-nest patients
	B. Special populations (e.g. elderly, children, pregnant, liver/kidney-impaired)
	C. Economically disadvantaged
	D. Patients suffering from adverse reactions
	E. Patients needing test report interpretations
	F. Frequent drug collectors (>20 times/year)
	G. Patients with ≥2 chronic diseases
	H. Patients with any chronic diseases
	I. Patients on ≥5 medications

Items	Answers
	J. High-risk drug users (e.g., psychotropic drugs, hormones, injections, and inhalants)
	K. Patients under contract with family physicians
	L. All patients
Q13: It is essential to conduct pharmacist clinics.	A. Totally agree
	B. Agree
	C. Uncertain
	D. Disagree
	E. Totally disagree
Q14: You are confident in providing pharmacist clinics.	A. Totally agree
	B. Agree
	C. Uncertain
	D. Disagree
	E. Totally disagree
Q15: What is the optimal modality of pharmacist clinics?	A. Physician-guided traditional clinic
	B. Independent pharmacist clinic
	C. Joint physician-pharmacist clinic
	D. Multidisciplinary clinic involving physicians, pharmacists, nurses, and nutritionists
	E. Lectures/consultation sessions; no scheduled clinics
Q16: What is the optimal approach for pharmacist clinics?	A. Collaboration with research institutions
	B. Collaboration with medical institutions
	C. Collaboration with enterprises
	D. Collaboration with industry associations
	E. Independent operation
	F. Other
Q17: What is the optimal frequency for pharmacist clinics?	A. Daily
	B. 2-4 times per week
	C. Weekly
	D. Once every two weeks
	E. Monthly
	F. Other (please specify ____)
Q18: What is the optimal fee for pharmacist clinics, excluding medication costs?	A. Registration and pharmacy service fees
	B. Only registration fees determined by provider positions
	C. Only registration fees determined by institution levels
	D. No fee should be charged
Q19: Has your institution conducted pharmacist clinics?	A. Yes
	B. No (Skip to Question 21)
Q20: How frequently does your institution operate the pharmacist clinics?	A. Daily
	B. 2-4 times per week
	C. Weekly
	D. Once every two weeks
	E. Monthly
	F. Other (please specify ____)
Q21: What is the primary challenge in setting up pharmacist clinics?	A. Insufficient professionalism
	B. High outpatient workload
	C. Limited patient volume
	D. Lack of leadership attention
	E. Weak inter-department collaboration
	F. Space constraints
	G. Absence of fee collection mechanisms
	H. Low staff motivation
	I. No significant difficulties identified
